# Porcine transmissible gastroenteritis virus nonstructural protein 2 contributes to inflammation via NF-κB activation

**DOI:** 10.1080/21505594.2018.1536632

**Published:** 2018-10-29

**Authors:** Li Wang, Xinyuan Qiao, Sijia Zhang, Yue Qin, Tiantian Guo, Zhenye Hao, Li Sun, Xiaona Wang, Yanan Wang, Yanping Jiang, Lijie Tang, Yigang Xu, Yijing Li

**Affiliations:** a Department of Preventive Veterinary Medicine, College of Veterinary Medicine, Northeast Agricultural University, Harbin, Heilongjiang, China; b Northeastern Science Inspection Station, China Ministry of Agriculture Key Laboratory of Animal Pathogen Biology, Harbin, Heilongjiang, China; c College of Animal Science and Technology, Northeast Agricultural University, Harbin, Heilongjiang, China

**Keywords:** TGEV, Nsp2, inflammation, NF-κB, replication

## Abstract

Transmissible gastroenteritis virus (TGEV) infection causes acute enteritis in swine of all ages, and especially in suckling piglets. Small intestinal inflammation is considered a central event in the pathogenesis of TGEV infections, and nuclear factor-kappa B (NF-κB) is a key transcription factor in the inflammatory response. However, it is unclear whether NF-κB is crucial for inducing inflammation during a TGEV infection. Our results show that NF-κB was activated in swine testicular (ST) cells and intestinal epithelial cell lines J2 (IPEC-J2) cells infected with TGEV, which is consistent with the up-regulation of pro-inflammatory cytokines. Treatment of TGEV-infected ST cells and IPEC-J2 cells with the NF-κB-specific inhibitor caused the down-regulation of pro-inflammatory cytokine expression, but did not significantly affect TGEV replication. Individual TGEV protein screening results demonstrated that Nsp2 exhibited a high potential for activating NF-κB and enhancing the expression of pro-inflammatory cytokines. Functional domain analyzes indicated that the first 120 amino acid residues of Nsp2 were essential for NF-κB activation. Taken together, these data suggested that NF-κB activation was a major contributor to TGEV infection-induced inflammation, and that Nsp2 was the key viral protein involved in the regulation of inflammation, with amino acids 1–120 playing a critical role in activating NF-κB.

**Abbreviations:** TCID50: 50% tissue culture infectious dose; DMEM: Dulbecco’s Modified Eagle Medium; eNOS: Endothelial nitric oxide synthase; FBS: fetal bovine serum; IFA: Indirect immunofluorescence; IκB: inhibitor of nuclear factor kappa-B; IL: interleukin; IPEC-J2: intestinal epithelial cell lines J2; IKK: IκB kinase; Luc: luciferase reporter gene; mAbs: monoclonal antibodies; MOI: multiple of infection; Nsp: nonstructural protein; NF-κB: nuclear factor-kappa ; ORFs: open reading frames; PBS: phosphate-buffered saline; p65 p-p65: phosphorylated; RT-PCR: reverse transcription PC; SeV: Sendai virus; ST: swine testicular; TGEV: Transmissible gastroenteritis virus; TNF-α: tumor necrosis factor α; UV-TGEV: Ultraviolet light-inactivated TGEV; ZnF: zinc finger

## Introduction

The transmissible gastroenteritis virus (TGEV) is a contagious porcine enteropathogenic virus belonging to the family Coronaviridae, and the order Nidovirales [1]. It is an enveloped virus possessing a single-stranded, positive-sense RNA genome with a length of approximately 28.6 kb, with a 5′ cap and a 3′ polyadenylated tail [2]. The TGEV genome contains at least nine open reading frames (ORFs), including two large ORFs in the 5′-proximal two-thirds of the genome (ORF1a and ORF1b), which encode the viral replicase [2], while one-third of the genome encodes the four structural proteins, S, M, N, and E, and the three accessory proteins, 3a, 3b, and 7 [2]. Various breeds of pigs, regardless of age, could be infected by TGEV, with suckling piglets under two weeks of age being the most susceptible. Infected animals present with vomiting, dehydration, and severe diarrhea, with the mortality rate possibly reaching up to 100% [3]. TGEV replicates in enterocytes covering the villi of the small intestine, resulting in severe gastroenteritis in diseased piglets [4], which suggests that the inflammation of the small intestine might be an important event in the pathogenesis of TGEV infections.

A considerable amount of research indicates that coronavirus pathogenesis is closely associated with excessive induction of pro-inflammatory cytokines, which are mainly driven by the activation of at least one of the following pathways: ATF-1/jun, jun/fos, IRF-3, IRF-7, NF-AT, or NF-κB [5–8]. Among these pathways, coronaviruses most frequently activate the NF-κB signaling pathway, and play an important role in coronavirus pathogenesis through the mediation of pro-inflammatory cytokine and chemokine production [6–9].

The NF-κB signaling pathway is a conserved pathway that regulates a variety of physiological and pathological processes, including cell proliferation, survival, and differentiation; metabolic, inflammatory, and immune diseases; and cancer [10]. In mammals, the NF-κB family of transcription factors consists of five members, p65/RelA, RelB, cRel, p50, and p52. All NF-κB transcription factors possess a structurally conserved amino-terminal Rel-homology domain that contains the dimerization, nuclear localization, and DNA-binding domains [11]. In unstimulated cells, NF-κB dimers are mainly retained in the cytosol in an inactive form that results from interactions with inhibitor proteins of the inhibitor of nuclear factor kappa-B (IκB) family. Various signals, including cytokines, growth factors, microbial products, stress-inducing stimuli, and T cell receptor engagement could induce canonical NF-κB signaling [12]. Upon activation, the IκB kinase (IKK) complex phosphorylates IκB proteins, resulting in their ubiquitination and subsequent proteasomal degradation, thereby releasing NF-κB dimers from the cytoplasmic NF-κB-IκB complex and allowing them to translocate to the nucleus, where they regulate the expression of target genes [13].

Severe gastroenteritis is a significant clinical sign of TGEV infection, and it is now well-established that NF-κB is a key regulator of inflammation because of its ability to induce the transcription of pro-inflammatory genes such as tumor necrosis factor α (TNF-α), interleukin (IL) −1β, IL-6, and IL-8 [14,15]. In the current study, we found that TGEV infection induced the inflammatory response through NF-κB signal activation, but that NF-κB inhibition did not significantly affect TGEV replication. Screening of TEGV-encoded proteins demonstrated that Nsp2 acted as a major component that was responsible for inducing an inflammatory response, and that the N-terminal 120-amino-acid region formed a critical domain involved in NF-κB activation.

## Results

### TGEV infection activates NF-κB

**Figure 1.**
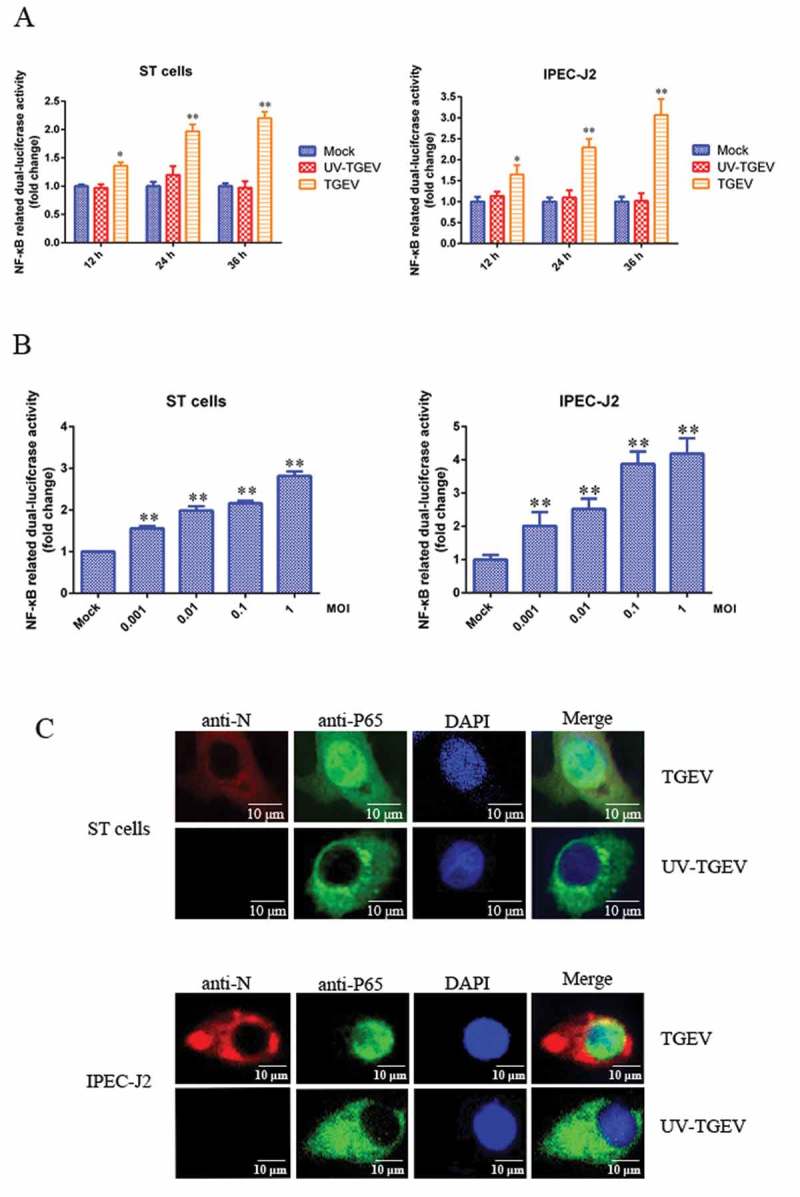
TGEV infection activated the NF-κB pathway. (a) ST cells and IPEC-J2 cells were transfected with pNF-κB-Luc and pRL-TK. At 12 h post-transfection, cells were mock-infected or infected with TGEV at an MOI of 0.1. At 12, 24, and 36 h after TGEV infection, cell extracts were prepared for carrying out luciferase reporter gene assays. (b) ST cells and IPEC-J2 cells were transfected with pNF-κB-Luc and pRL-TK. At 24 h post-transfection, cells were infected with TGEV at an MOI of 0.001, 0.01, 0.1, or 1. Cell extracts were prepared for luciferase reporter gene assays at 36 h post-infection. Results are representative of three independent experiments. Data are presented as mean ± SD. P values < 0.05 (*) and < 0.01 (**) were considered to be statistically significant and highly significant, respectively. (c) ST cells and IPEC-J2 cells were mock-infected or infected with TGEV at an MOI of 0.1. At 36 h post-infection, cells were fixed and analyzed with the mouse anti-N and Rabbit anti-P65 antibodies, followed by TRITC-conjugated goat anti-mouse secondary antibody (red) and FITC-conjugated goat anti-rabbit secondary antibody (green). Cellular nuclei were stained with DAPI. Nuclear translocation of p65 was observed under a fluorescence microscope.

To determine whether TGEV infection had regulatory effects on the NF-κB signaling pathway, ST cells and IPEC-J2 cells were transiently transfected with the pNF-κB-Luc reporter plasmid along with pRL-TK, which was used as an internal reference. After 12 h, cells were mock-infected or infected with TGEV or UV-TGEV. At various times post-infection, the luciferase activity of TGEV-infected cells was significantly higher than that of the UV-TGEV-infected group and the mock-infected group (Figure 1(a)). To confirm whether there was a direct link between the viral-infection dose and NF-κB activity, ST cells and IPEC-J2 cells transfected with pNF-κB-Luc and pRL-TK were infected with TGEV at different titers. We found that TGEV infection enhanced NF-κB-dependent luciferase expression in a dose-dependent manner (Figure 1(b)). To further confirm the activation of the NF-κB signaling pathway during TGEV infection, the translocation of p65 was monitored in ST cells and IPEC-J2 cells infected with TGEV by fluorescence microscopy. As shown in Figure 1(c), the p65 protein accumulated in the cytoplasm of mock-infected cells, while it was translocated into the nucleus of TGEV-infected cells. These data are consistent with NF-κB signaling pathway activation in ST and IPEC-J2 cells following TGEV infection.

### TGEV infection induces inflammatory response via the NF-κB signaling pathway

**Figure 2.**
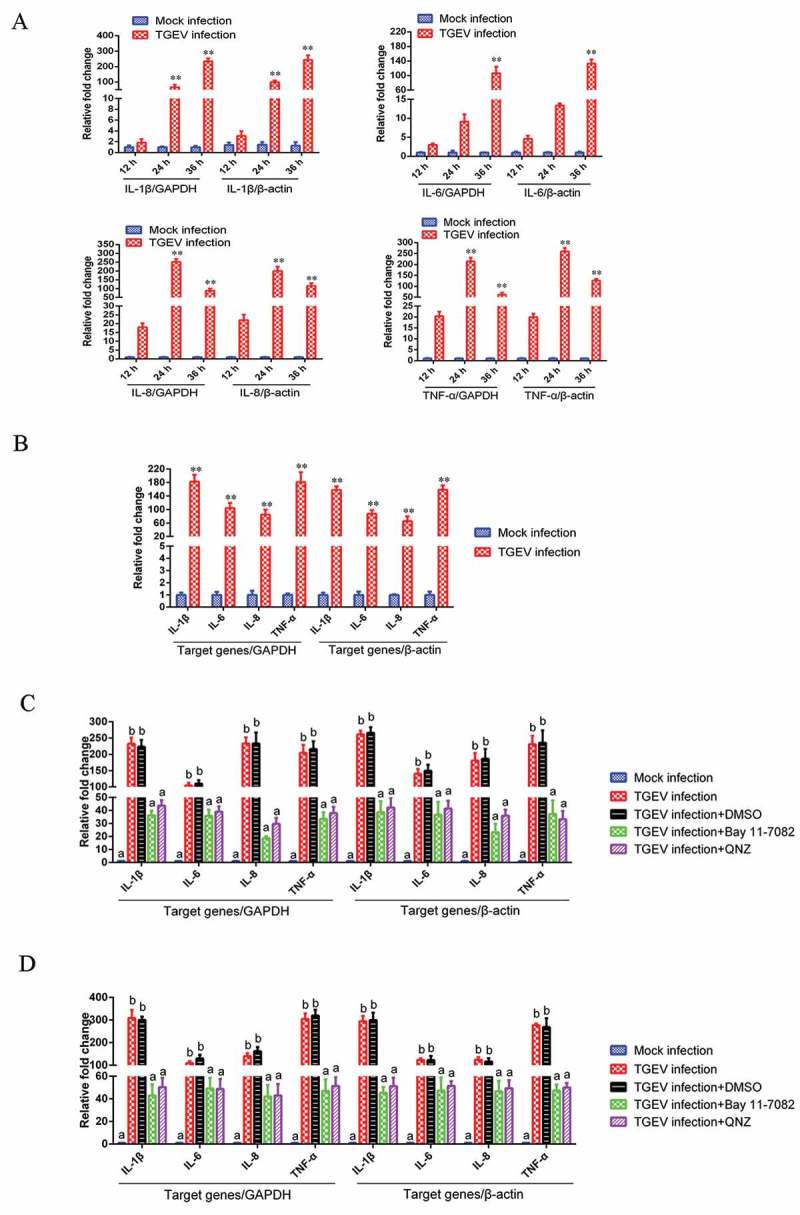
TGEV infection induced inflammation through the activation of the NF-κB pathway. (a) Effects of TGEV on the NF-κB-regulated mRNA expression of IL-1β, IL-6, IL-8, and TNF-α in ST cells. ST cells were mock-infected or infected with TGEV at an MOI of 0.1. Expression levels of mRNA for IL-1β, IL-6, IL-8, and TNF-α were measured by real-time RT-PCR at 12, 24, and 36 h after TGEV infection. GAPDH and β-actin were used as internal reference genes, respectively. (b) Effects of TGEV on NF-κB-regulated mRNA expression of IL-1β, IL-6, IL-8, and TNF-α in IPEC-J2 cells. IPEC-J2 cells were mock-infected or infected with TGEV at an MOI of 0.1. Expression levels of mRNA for IL-1β, IL-6, IL-8, and TNF-α were measured by real-time RT-PCR at 36 h after TGEV infection. GAPDH and β-actin were used as internal reference genes, respectively. Values are the mean ± SD of three independent tests. *P* values < 0.05 (*) and < 0.01 (**) compared with mock infection group. (c) ST cells and (d) IPEC-J2 cells were infected with TGEV at an MOI of 0.1. Bay 11–7082 or QNZ was added to the media at 1 h post-infection at a final concentration of 5 μM or 10 μM, respectively. Cells treated with DMSO served as negative controls. The mRNA expression levels of IL-1β, IL-6, IL-8, and TNF-α were measured at 36 h post-infection via real-time RT-PCR. GAPDH and β-actin were used as internal reference genes, respectively. Different letters indicate significant differences (*P *< 0.01).

To explore whether TGEV infection-induced inflammation was regulated through the NF-κB signaling pathway, the expression of NF-κB-regulated pro-inflammatory genes in TGEV-infected ST cells and IPEC-J2 cells was measured using real-time reverse transcription PCR (RT-PCR). In order to improve the accuracy of the results, two internal reference genes, GAPDH and β-actin, were used in RT-PCR respectively. The results of RT-PCR showed that IL-1β, IL-6, IL-8, and TNF-α mRNA levels were significantly elevated following TGEV infection, no matter which gene was used as the internal reference (Figure 2(a,b)). Moreover, increased mRNA expression of Arginase and eNOS was detected in TGEV infected cells (Supplementary material Figure S1). Bay 11–7082 and QNZ, which specifically block NF-κB activation, profoundly decreased the expression of pro-inflammatory genes in TGEV infected ST cells (Figure 2(c)) and IPEC-J2 cells (Figure 2(d)). Therefore, it was inferred that the TGEV infection-induced inflammatory response was associated with the NF-κB signaling pathway activation.

### TGEV replication was not significantly affected by the NF-κB signaling pathway

**Figure 3.**
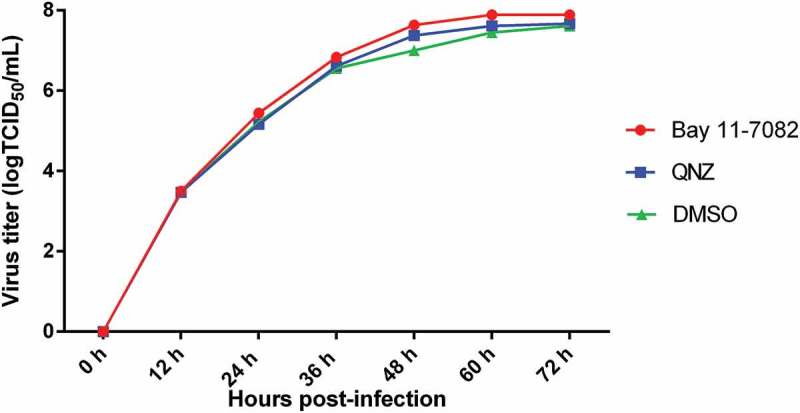
The effects of the NF-κB signaling pathway on TGEV replication. ST cells were infected with TGEV at an MOI of 0.1. After 1 h, Bay 11–7082 or QNZ was added to the media at a final concentration of 5 or 10 μM, respectively. Cells treated with DMSO served as a negative control. At 12, 24, 36, 48, 60, and 72 h post-infection, the viral titers in the media were determined by the TCID50 values. Results are representative of three independent experiments. Data are presented as means ± SD. *P* values that were < 0.05 (*) were considered to be statistically significant.

To determine the role of the NF-κB signaling pathway in the replication of TGEV, the NF-κB-specific inhibitor Bay 11–7082 and QNZ were used to inhibit NF-κB activation in ST cells infected with TGEV, respectively; viral titers were titrated by 50% tissue culture infectious dose (TCID50) assays at different time-points post-infection. The viral growth kinetics during NF-κB inhibition was assessed. TGEV exhibited a slightly slower replication rate in DMSO-treated cells than in the NF-κB-specific inhibitor-treated cells, but the difference in replication rates was insignificant (Figure 3). These results suggest that NF-κB signaling pathway activation has no significant effect on TGEV replication.

### Nsp2 is primarily responsible for the TGEV-induced activation of the NF-κB signaling pathway

**Figure 4.**
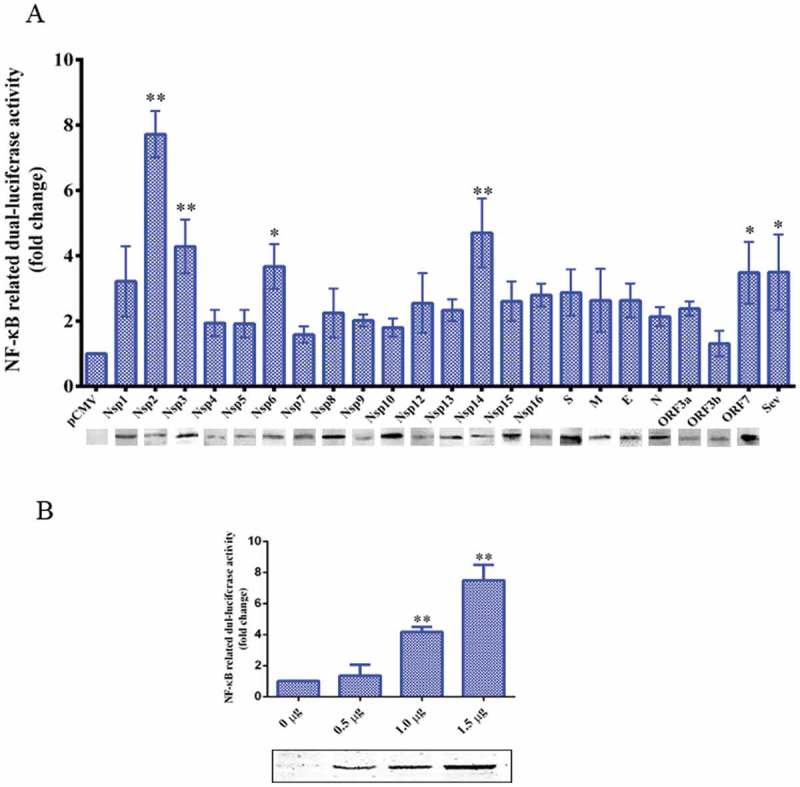
TGEV Nsp2 activated NF-κB. (a) ST cells were co-transfected with pNF-κB-Luc, pRL-TK, and the indicated expression plasmid encoding the TGEV protein, or truncated segments. At 36 h post-transfection, cells were harvested and analyzed by western blotting using the anti-HA antibody and cell extracts were prepared for luciferase reporter gene assays. *P* values < 0.05 (*) and < 0.01 (**) were considered to be statistically significant and highly significant, respectively.(b) Increasing quantities of Nsp2 expression plasmids (0 μg, 0.5 μg, 1 μg, and 1.5 μg) were co-transfected with pNF-κB-Luc and pRL-TK into ST cells. Cells were harvested and analyzed by western blotting using the anti-HA antibody and luciferase activity measurement at 36 h post-transfection. Results are representative of three independent experiments. Data are presented as mean ± SD values. *P* values < 0.05 (*) and < 0.01 (**) were considered statistically significant and highly significant, respectively.

To identify the TGEV proteins that might play a role in the activation of the NF-κB signaling pathway, the TGEV proteins were screened for their capacity to activate NF-κB using a luciferase reporter assay. We found that all TGEV proteins except Nsp7 and ORF3b could activate the NF-κB signaling pathway upto a varied extent, and that Nsp2 exhibited the strongest capability for activation (Figure 4(a)). To confirm the activation of the NF-κB signaling pathway by Nsp2, ST cells were transfected with increasing amounts of Nsp2-expressing plasmids. The luciferase activity levels in the transfected ST cells correlated with the increased expression of Nsp2 (Figure 4(b)). These results indicated that Nsp2 is the major viral protein responsible for the activation of the NF-κB signaling pathway during TGEV infection.

### Nsp2 promoted IκBα degradation and p65 nuclear translocation

**Figure 5.**
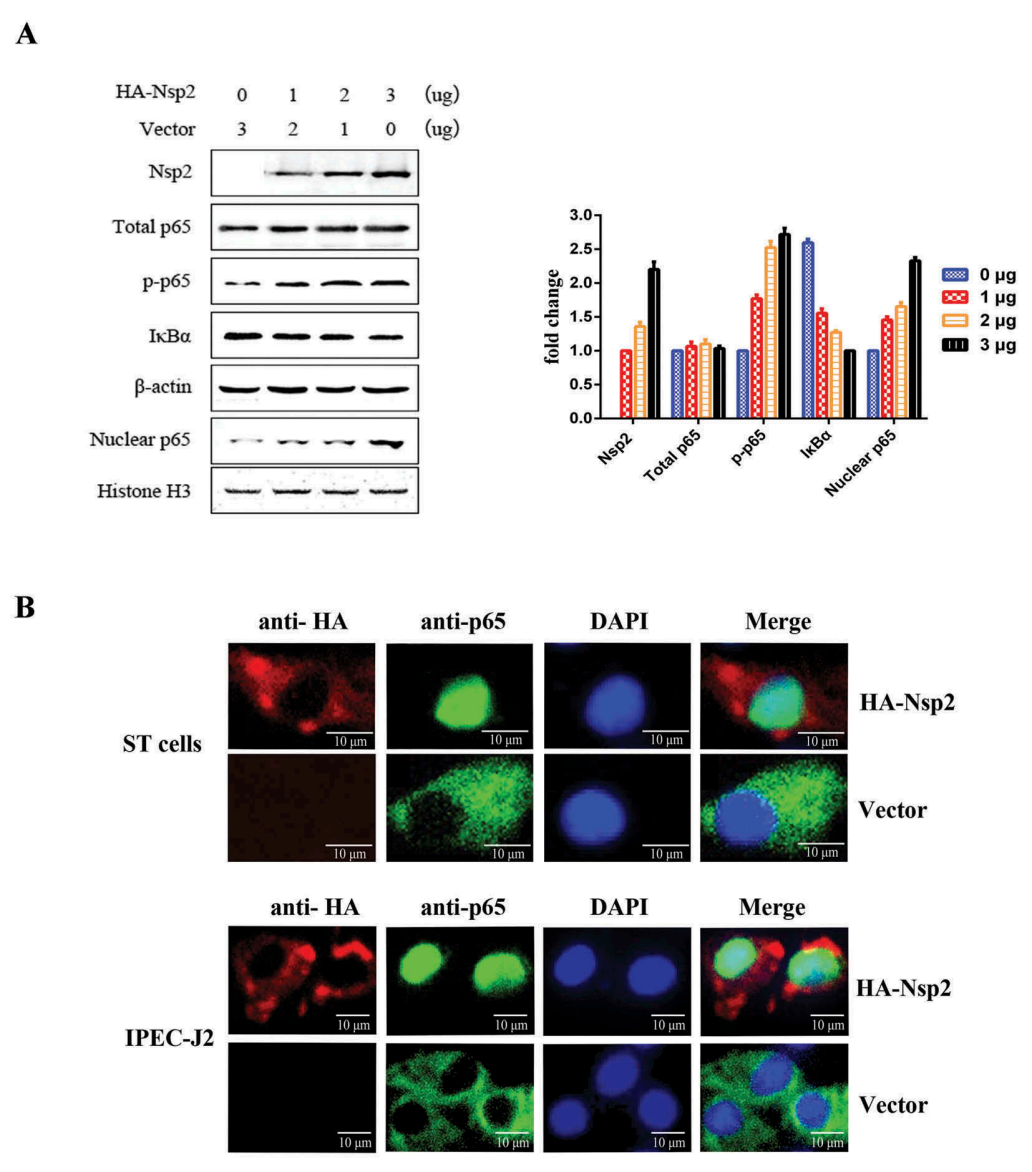
TGEV Nsp2 induced the degradation of IκBα and the nuclear translocation of p65. (a) ST cells were transfected with 0 μg, 1 μg, 2 μg, or 3 μg of TGEV Nsp2 expression plasmids, as indicated. The total amount of transfected DNA was kept equal by adding appropriate amounts of the empty vector. At 36 h post-transfection, cytoplasmic and nuclear extracts were prepared and subjected to western blot analysis using anti-HA antibody, antibodies specific for endogenous IκBα, p65, and phosphorylated p65 (p-p65), using β-actin and histone H as the controls. The right panel represents the quantification of the bands by densitometry, corrected by the amount of β-actin or histone H3.(b) Nsp2-HA was transiently expressed in ST cells and IPEC-J2 cells，respectively. At 36 h post-transfection, the cells were fixed, permeabilized, and stained with antibodies to HA and endogenous p65, followed by TRITC-conjugated goat anti-mouse secondary antibody (red) and FITC-conjugated goat anti-rabbit secondary antibody (green). Nuclei were counterstained with DAPI (blue). The cells were imaged under a fluorescence microscope.

The degradation of IκBα following its phosphorylation by the IKK complex and nuclear translocation of p65 are considered to be the hallmarks of NF-κB signaling pathway activation [16]. ST cells were transfected with increasing amounts of Nsp2-expressing plasmids and western blot analyzes were used to investigate IκBα expression levels, phosphorylation, and the nuclear translocation of p65 in cell lysates. The results obtained after western blotting showed that the expression of Nsp2 had no significant effects on the total amount of p65; however, the level of phosphorylated p65 (p-p65) and nuclear p65 increased markedly, and the level of IκBα decreased significantly (Figure 5(a)). To further confirm the nuclear translocation of NF-κB after Nsp2 expression, ST cells and IPEC-J2 cells that expressed the HA-Nsp2 fusion protein were used to monitor p65 with IFA. The IFA data showed that endogenous p65 accumulated in the cytoplasm of ST cells and IPEC-J2 cells transfected with the empty vector, while it was translocated to the nucleus in Nsp2-expressing cells (Figure 5(b)). These results demonstrate that the Nsp2-induced activation of NF-κB was characterized by IκBα degradation and p65 nuclear translocation.

### Nsp2 contributed to the increased expression of NF-κB-regulated pro-inflammatory genes induced by TGEV infection

**Figure 6.**
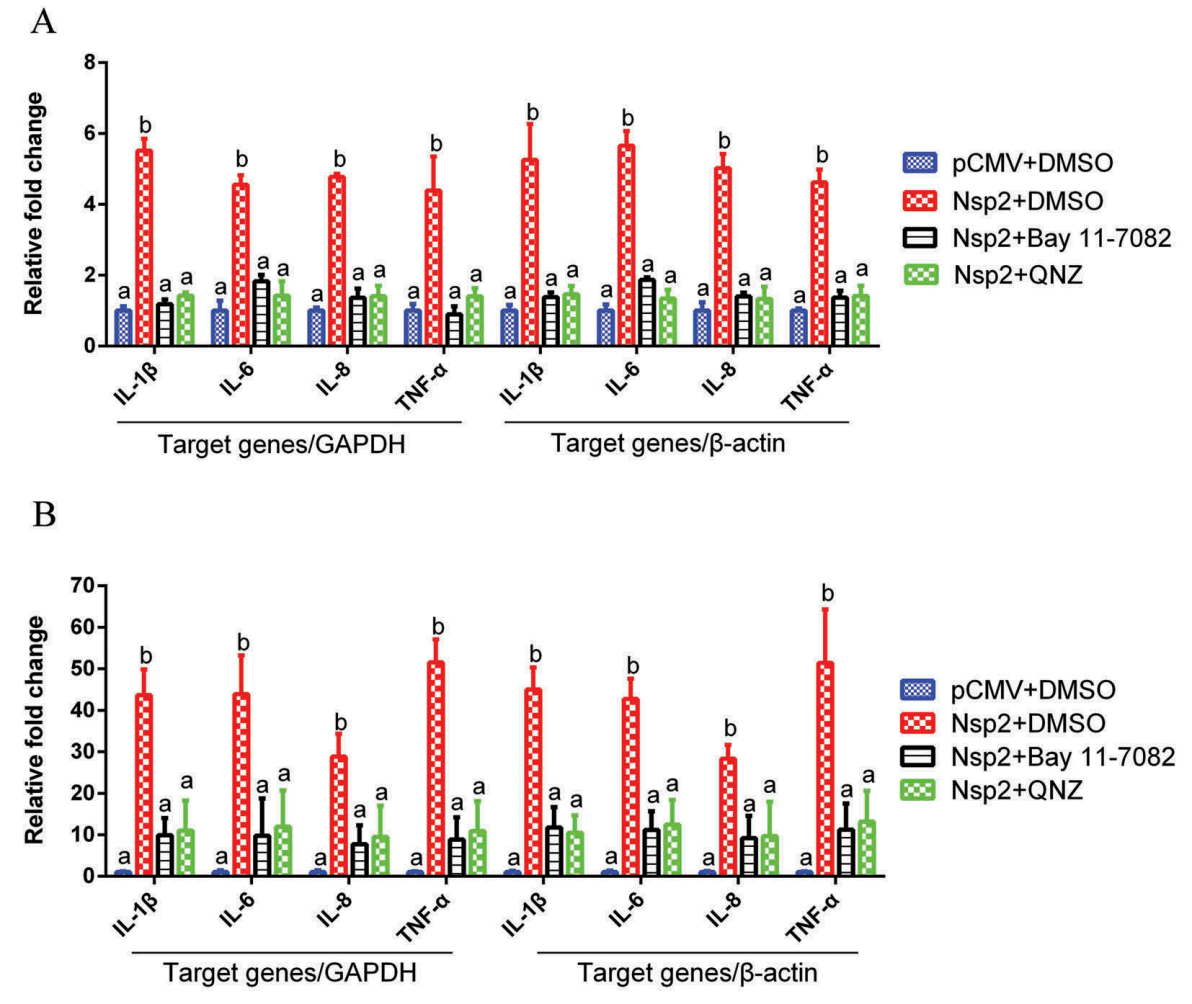
Nsp2 expression enhanced the expression of NF-κB-regulated pro-inflammatory genes. ST cells (a) and IPEC-J2 (b) cells were transfected with plasmid encoding Nsp2 or the pCMV vector, respectively. After 12 h, Bay 11–7082 was added to the culture media to a final concentration of 5 μM. Cells transfected with the pCMV plasmid or treated with DMSO served as negative controls. The mRNA expression levels of IL-1β, IL-6, IL-8, and TNF-α were measured at 36 h post-transfection via real-time RT-PCR. GAPDH and β-actin were used as internal reference genes, respectively. Results are representative of three independent experiments. Data are presented as mean ± SD values. Different letters indicate significant differences (*P *< 0.01).

To further determine whether Nsp2 contributed to the enhancement of NF-κB-regulated pro-inflammatory gene expression, which was induced by TGEV infection, ST cells and IPEC-J2 cells were transfected with the Nsp2 expression plasmid respectively, and treated with NF-κB-specific inhibitor (Bay 11–7082 or QNZ) at 12 h post-transfection. The results showed that Nsp2 enhanced the expression of NF-κB-regulated pro-inflammatory genes IL-1β, IL-6, IL-8, and TNF-α. However, in the NF-κB-specific inhibitor-treated group, Nsp2 exhibited a reduced ability to upregulate the expression of these genes, as compared to that observed with the DMSO-treated group (Figure 6). These findings suggest that Nsp2 played a critical role in stimulating pro-inflammatory gene expression, which was induced via the NF-κB signaling pathway during TGEV infection. Additionally, increased mRNA expression of Arginase and eNOS was detected in Nsp2-expressing cells (Supplementary material Figure S2).

### N-terminal 120 amino acid residues of TGEV Nsp2 were responsible for activating the NF-κB signaling pathway

**Figure 7.**
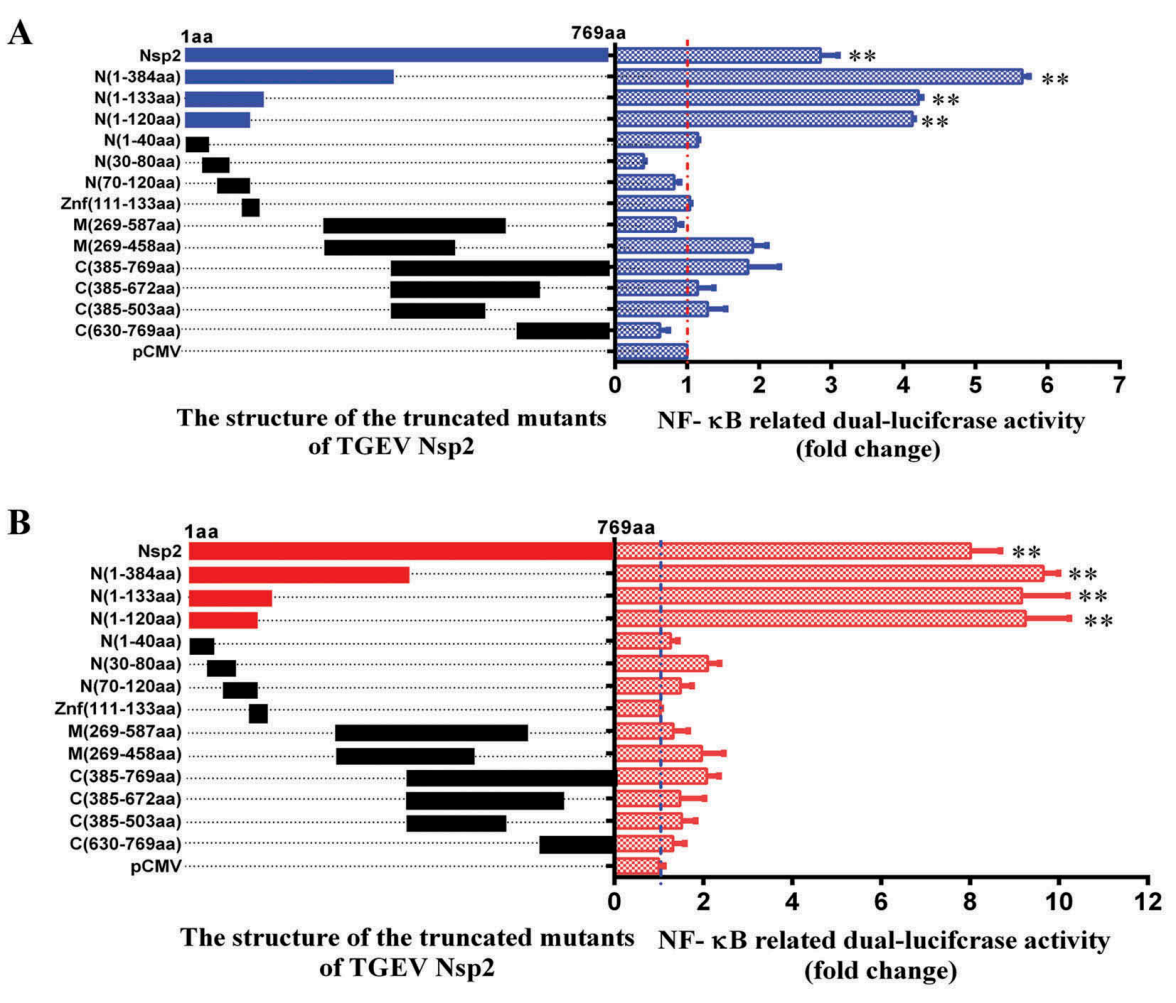
The amino acids 1–120 of Nsp2 were responsible for the activation of the NF-κB signaling pathway. (a) ST cells and (b) IPEC-J2 cells were co-transfected with pNF-κB-Luc, pRL-TK, and the distinct truncated mutants of Nsp2 expression plasmids (schematic diagrams of the structure of the TGEV-truncated Nsp2 mutants are on the left of figure a and b). The empty pCMV-HA vector was used as a control. Cells were harvested and analyzed for luciferase activity at 36 h post-transfection. Results are representative of three independent experiments. Data are presented as mean ± SD values. *P* values < 0.05 (*) and < 0.01 (**) were considered statistically significant and highly significant, respectively.

In efforts to map the region within Nsp2 responsible for activating the NF-κB signaling pathway, we constructed a series of truncated mutants of Nsp2 (Figure 7, left). ST cells (Figure 7(a)) and IPEC-J2 cells (Figure 7(b)) that were co-transfected with the mutant constructs and the pNF-κB-luc reporter plasmid were screened for luciferase activity to identify the functional segments responsible for pathway activation. As shown in Figure 7 (right), transfection of cells with vectors carrying either the full-length or truncated mutants of Nsp2 containing a gene fragment encoding at least amino acids 1–120 of Nsp2 caused a significant enhancement in NF-κB luciferase activity. However, the truncated mutants that lacked amino acids 1–120 of Nsp2 failed to activate the NF-κB reporter gene. The SMART database indicates that there is a zinc finger (ZnF) domain located on the N-terminal end of Nsp2. Because of this, we wanted to investigate whether the ZnF had any effect on the NF-κB signaling pathway. To test this hypothesis, we constructed an expression plasmid containing the ZnF domain, and analyzed its capacity to stimulate the pathway. However, the results of these experiments showed that the ZnF domain did not affect NF-κB activation. Consistent results were obtained in ST cells (Figure 7(a)) and IPEC-J2 (Figure 7(b)) cells. Overall, our results suggested that amino acids 1–120 of Nsp2 composed the critical domain that was responsible for activating the NF-κB signaling pathway.

## Discussion

Coronaviruses constitute a significant threat to both human and animal health. Pathogenic inflammation is activated by a coronavirus infection, which might result in the death of the host [17,18]. NF-κB is highly activated in diverse coronaviruses, including the severe acute respiratory syndrome coronavirus (SARS-CoV), Middle East respiratory syndrome coronavirus (MERS-CoV), human coronavirus, murine coronavirus, porcine epidemic diarrhea virus (PEDV), and infectious bronchitis virus (IBV) [6,19–22]. The NF-κB-induced inflammation in these coronavirus infections plays an important role in pathogenesis and disease development.

Porcine TGEV is an enteropathogenic coronavirus that damages the small intestine and causes severe intestinal inflammation in piglets [23], and increased expression levels of the proinflammatory cytokines IL-1β, IL-6, and TNF-α have been detected in the jejunum [24]. Although TGEV-induced inflammation has been well-established, its exact underlying molecular mechanism is not understood. Previous studies show that NF-κB was activated in TGEV-infected ST and porcine kidney (PK)-15 cells [7,8]. Considering that the small intestine was the main target organ attacked by TGEV and that IPEC-J2 cells, a spontaneously immortalized cell line from the porcine intestine, are likely much more representative of porcine physiology and the true state of virus infection in animals [25,26], IPEC-J2 cells were used in current study in addition to ST cells. Previous studies showed that IPEC-J2 cells infected with TGEV displayed increased expression levels of proinflammatory cytokines, including IL-1β, IL-6, IL-8, TGF-β, and TNF-α [27,28]. The current study further demonstrated that TGEV could activate NF-κB in IPEC-J2 cells, by using a luciferase reporter gene assay and IFA. In addition, the expression of pro-inflammatory genes induced by the TGEV infection was associated with NF-κB activation. In another similar phenomenon, treatment with the NF-κB-specific inhibitor significantly decreased TGEV-induced proinflammatory cytokine production, as described in Ding’s study [29]. The above results suggested that intestinal inflammation during TGEV infection resulted mainly because of NF-κB activation.

Inflammation is an innate immune defense mechanism that is induced in response to physical, physiological, and/or oxidative stresses, and NF-κB functions as a crucial coordinator of inflammatory and immune response [10]. In addition, NF-κB plays a critical role in many cellular processes, including cell proliferation, survival, and differentiation [30]. Some viruses utilize NF-κB modulation to evade the innate immune response of the host that limit replication by killing infected cells, as well as to enhance viral replication and exploit the anti-apoptotic properties of NF-κB, or trigger apoptosis for increasing the spread of viruses [31]. To explore whether NF-κB activation influences TGEV replication, we used the NF-κB-specific inhibitors to inhibit NF-κB activation in cells infected with TGEV. To improve the accuracy of the results, two NF-κB-specific inhibitors, Bay 11–7082 and QNZ, were used in this study. TGEV exhibited a slightly faster growth rate in NF-κB-specific inhibitor-treated cells than in DMSO-treated cells, but it did not reach a significant level. Consistent results were obtained in the different inhibitor-treated groups. These results suggested NF-κB signaling pathway activation had no remarkable effects on TGEV replication, which was consistent with the results of the previous study [29]. Conversely, other researchers reported that NF-κB inhibition leads to the suppression of TGEV replication [7]. It should be noted that Yang et al [7] and our group employed different NF-κB inhibitors that inhibited NF-κB activation through different pathways. Multiple associations between signaling pathways or networks might have orchestrated NF-κB activation [32]; a previous study showed that TGEV induced NF-κB activation through RLR-mediated signaling in PK-15 cells [29] and Yang’s results also indicate that there are at least two convergent pathways associated with NF-κB activation that are induced by TGEV infections in ST cells, IKK-2_IκBα/P65 and JAK2 [7]. Additionally, the amount of NF-κB inhibitors may be one factor affecting TGEV replication. Further studies are required to understand this in more detail.

Coronaviruses are the largest of all RNA viruses, with positive-strand RNA genomes that encode a series of non-structural proteins, structural proteins, and accessory proteins. The roles of many coronavirus proteins in the regulation of inflammation have been identified. For example, the S protein of the SARS coronavirus stimulates murine macrophages to produce pro-inflammatory IL-6 and TNF-α by activating NF-κB [33], while the absence of the SARS E protein results in a reduced expression of pro-inflammatory cytokines [6]. In addition, the accessory proteins 3a/X1 and 7a/X4 of SARS are capable of activating both NF-κB and JNK, and significantly enhance IL-8 promoter activity [9]. Human coronavirus OC43 (HCoV-OC43) encodes an accessory protein ns12.9, which is involved in the inflammatory response during an HCoV-OC43 infection [34]. PEDV-encoded N and S proteins are responsible for the up-regulation of IL-6 and IL-8 expression [35,36]. In the current study, results obtained after screening TGEV-encoded proteins demonstrated that Nsp2 was the most potent NF-κB activator and that it induced pro-inflammatory IL-1β, IL-6, IL-8, and TNF-α production, mainly by the activation of the NF-κB signaling pathway. Nsp3, Nsp6, Nsp14, and ORF7 were also found to be potential NF-κB activators, in addition to Nsp2. Nsp14 has been identified as a key inducer of IFN-β production in HEK-293T cells and PK-15 cells and has been found to act through the activation of NF-κB [8]; in addition, a specific mutation within Nsp14 led to a reduction in the expression of IFN-β, TNF, and interferon-stimulated genes [37]. According to our results, 4NF-κB activation was also observed in Nsp14-transfected ST cells. However, the potential of Nsp14 in inducing NF-κB activation in ST cells is weaker than that of Nsp2. Additionally, the potential of Nsp2 in inducing NF-κB activation in IPEC-J2 cells was also be confirmed in this study. There are multiple possible reasons for the discrepancies, the different kinds of cells were probably the main reason. Relatively fewer studies have investigated the effects of other TGEV-encoded proteins.

Biological software has predicted that TGEV Nsp2, an 85-kDa protein encoded by the replicase gene, is composed of several functional domains, including a ZnF structure. ZnFs are independently folded domains containing approximately 30 amino acids, which are maintained by a zinc ion, and coordinated through cysteine and histidine residues in different combinations [38]. They are implicated in the regulation of several cellular processes, including replication, signal transduction, cell proliferation, and apoptosis [38,39]. Interestingly, the ZnF of TGEV Nsp14 is reported to potentially play a role in modulating the expression of IFN-β, TNF-α, and interferon-stimulated genes in ST cells [37]. However, it is still unclear whether the ZnF of TGEV Nsp2 is associated with NF-κB activation. Our data suggest that the ZnF of TGEV Nsp2 was unrelated to the activation of NF-κB. Even with the uncertainty regarding the effect of the ZnF, our analyzes, which were performed using truncated mutants allowed us to map the key domain responsible for activating NF-κB to the 120 amino acids of the N-terminal end of the Nsp2 protein. Although the above experiments were conducted *in vitro*, IPEC-J2 cells are morphologically differentiated cells derived from the small intestine of young piglets, which can be used as a well-defined *in vitro* model with comparable intestinal physiology [25–28]. Based on these results, we predicted that Nsp2 could promote intestinal inflammation in TGEV-infected piglets, and the Nsp2-induced inflammation may cause damage of intestine barrier which would benefit the spread of TGEV to deeper intestine tissue.

In summary, our data demonstrated that TGEV infections induced pro-inflammatory cytokine production through NF-κB activation, and that amino acids 1–120 of Nsp2 were the most significant among the TGEV-encoded proteins for activation of NF-κB, indicating that the inflammation induced by the expression of Nsp2 during TGEV infection via NF-κB activation might play a crucial role in the pathogenesis of TGEV.

## Materials and methods

### Viruses, cells, and reagents

The TGEV TH-98 strain was isolated from the intestinal tract contents of TGEV-infected piglets in the Heilongjiang province of China (GenBank accession number: KU729220. 1); ST cells were obtained from the American Type Culture Collection (ATCC, CRL-1746); IPEC-J2 cells were a kind gift from Professor Xinna Ge (China Agricultural University); Sendai virus (SeV) cultures were maintained in our laboratory. The ST cells and IPEC-J2 cells were cultured in Dulbecco’s Modified Eagle Medium (DMEM) (Gibco, USA) supplemented with 10% fetal bovine serum (FBS) (Gibco) at 37°C in a humidified 5% CO2 incubator, and was used to amplify TGEV. Ultraviolet light-inactivated TGEV (UV-TGEV) were developed by irradiating TGEV stocks under ultraviolet light at a dose of 100 mJ/cm^2^. Mouse anti-β-actin and mouse anti-HA monoclonal antibodies (mAbs) were purchased from Sigma (USA). Rabbit polyclonal antibodies against HA were purchased from Thermo Fisher Scientific (USA). Mouse mAbs against NF-κB, p65, and IκBα, and rabbit mAbs against phospho-NF-κB p65 were obtained from Cell Signaling Technology (USA).

### Plasmids

The luciferase reporter plasmid pNF-κB-luc containing κB binding motifs and the luciferase reporter gene (Luc), and the control plasmid pRL-TK were purchased from Beyotime Biotechnology (China). The plasmid pCMV-HA was purchased from Clontech (USA). The expression plasmids of TGEV-encoded proteins used in this study were constructed by RT-PCR amplification from the genomic RNA of the TGEV strain TH-98 (GenBank accession number: KU729220. 1) and cloned to the pCMV-HA vector. The primers used in vector construction are shown in Table 1.

**Table 1.** Primers used for vector construction.

Gene name	Forward primer sequence (5ʹ-3ʹ)	Reverse primer sequence (5ʹ-3ʹ)	Position (bp)
Nsp1	**GGCCATGGAGGCC**ATGAGTTCCAAACAATTCAAG	**CTCGAGA**CCTCTGCCAGTGCGAGC	289–618
Nsp2	**GGCCATGGAGGCC**GCCATATATGTTGATCAATAC	**CTCGAGA**CCACCCATTTTATTATACATTC	619–2925
Nsp3	**GGCCATGGAGGCC**GGTGACAAAACTGTCTCAT	**CTCGAG**TGAACCACTTTTTGGAGACAC	2926–7455
Nsp4	**GGCCATGGAGGCC**GGCTTTTTCGATGTAATTAC	**CTCGAG**TGACTGAAGTGTAGAATTAACACTAAC	7456–8925
Nsp5	**GGCCATGGAGGCC**GGTTTGCGGAAAATGGCACAG	**CTCGAG**CTGAAGATTTACACCATACATTTGC	8926–9828
Nsp6	**GGCCATGGAGGCC**GCTGGTAAAGTAAAATCTTTC	**CTCGAG**CTGTACAGTTGAAATTTTGATG	9829–10710
Nsp7	**GGCCATGGAGGCC**TCAAAACTTACAGAGATGAAATG	**CTCGAG**CTGGAGTATGGTGGTGTTCTC	10711–10959
Nsp8	**GGCCATGGAGGCC**AGTGTGGCTTCAGCTTATGC	**CTCGAG**CTGAAGCTTTGTGGTTCTCTC	10960–11544
Nsp9	**GGCCATGGAGGCC**AACAATGAAATTATGCCAGG	**CTCGAG**CTTGCAGACGAACTGTTGCACC	11545–11877
Nsp10	**GGCCATGGAGGCC**GCTGGTAAACCCACTGAACATC	**CTCGAG**TTGATCAACAGTAAAACTCTGC	11878–12300
Nsp12	**GGCCATGGAGGCC**AAGTTATTTAAACGAGTGC	**CTCGAG**TTGCAAGACAGTGGACTTTTC	12300–15068
Nsp13	**GGCCATGGAGGCC**GCTGCAGGCATGTGTGTAG	**CTCGAG**AGGTTTTGCTTGTAAACCAATC	15069–16874
Nsp14	**GGCCATGGAGGCC**GAAACTTGTGGTTTATTTAAAG	**CTCGAG**CTGAAGTGCTTTGCTATTAAC	16875–18422
Nsp15	**GGCCATGGAGGCC**AGTCTAGAAAATGTGGCTTTTAATG	**CTCGAG**TTGGAGTTGTGGATAGAAGG	18423–19439
Nsp16	**GGCCATGGAGGCC**TCTGCTGAATGGAATCCCGGC	**CTCGAG**TCATGGTGTGTTAACGAAGTGG	19440–20342
S	**GGCCATGGAGGCC**ATGAAAAAACTATTTGTGG	**GCGGCCGC**TTAATGGACGTGCACTTTTTC	20339–24688
ORF3a	**GGCCATGGAGGCC**ATGGACATTGTCAAATCC	**CTCGAG**TTAAACAACTATATGACTATTG	24827–25042
ORF3b	**GGCCATGGAGGCC**ATGATTGGTGGACTTTTTC	**CTCGAG**CTAGGAAACGTCATAGGTATGGTC	25116–25850
E	**GGCCATGGAGGCC**ATGACGTTTCCTAGGGCATTG	**CTCGAG**TCAAGCAAGGAGTGCTCCATC	25837–26085
M	**GGCCATGGAGGCC**ATGAAGATTTTGTTAATATTAG	**CTCGAG**TTATACCATATGTAATAATTTTTC	26096–26884
N	**GGCCATGGAGGCC**ATGGCCAACCAGGGACAACG	**CTCGAG**TTAGTTCGTTACCTCATCAATTATC	26897–28045
ORF7	**GGCCATGGAGGCC**ATGCTCGTCTTCCTCCATGC	**CTCGAG**TTACATTAATGTGACTAACAATCTG	28051–28287

The restriction enzyme cutting sites are highlighted in bold.

### Transfection and reporter gene assays

ST cells or IPEC-J2 cells seeded into 24-well plates at a concentration of 0.5–1.0 × 10^5^ cells/mL were cotransfected with the pNF-κB-luc reporter plasmid (0.5 μg/well), control plasmid pRL-TK (0.025 μg/well), pCMV-HA expression plasmids containing TGEV proteins, or empty pCMV-HA plasmids using lipofectamine LTX and Plus^TM^ Reagent (Invitrogen). For infection studies, ST cells or IPEC-J2 cells were infected with TGEV or SEV or mock-infected 12 h post transfection. At indicated time points, cell extracts were harvested, and the firefly luciferase and Renilla luciferase activities were detected using the Dual-Luciferase® Reporter Assay System (Promega, USA), according to the manufacturer’s protocol. All reporter gene assays were repeated at least three times. Data are presented as the mean ± SD values.

### Indirect immunofluorescence (IFA)

At 36 h post-infection or post-transfection, ST cells or IPEC-J2 cells were fixed with 4% paraformaldehyde for 30 min and permeabilized with 0.2% Triton-100 in phosphate-buffered saline (PBS) for 10 min at room temperature. After three PBS washes, the cells were blocked with 5% BSA in PBS for 1 h at 37°C. The samples were incubated separately with primary antibodies for 1 h at room temperature, and treated with FITC-labeled goat anti-mouse IgG and/or TRITC-labeled goat anti-rabbit IgG for 1 h, which was followed by treatment with DAPI for 15 min at room temperature. Following three rinses, the samples were visualized using a Leica 2000 fluorescence microscope.

### Western blotting

The cells were washed with ice-cold PBS and treated with RIPA lysis buffer containing 1 mM PMSF (Beyotime, China). The cell lysates were sonicated and then centrifuged at 12,000 rpm at 4°C for 20 min. Protein concentrates were quantitated using a BCA protein assay kit (Beyotime, China). Protein samples were mixed with 5X SDS loading buffer and boiled for 10 min before being separated by SDS-PAGE. The electrophoretically resolved proteins were transferred onto PVDF membranes (Millipore, USA) and blocked with 5% nonfat dry milk at room temperature for 1 h, and the membranes were incubated with the indicated primary antibodies overnight at 4°C; they were then incubated with HRP-conjugated secondary antibodies at room temperature for 1 h. The ECL Reagent (Millipore, USA) was used for chemiluminescent detection of HRP-labeled proteins, according to the manufacturer’s instructions.

### Real-time reverse transcription polymerase chain reaction (RT-PCR)

ST cells were infected with TGEV at a multiple of infection (MOI) of 0.1, or mock-infected. Total RNA was extracted from the infected cells using TRIzol reagent (Invitrogen, USA) at 12 h, 24 h, and 36 h post-infection, and reverse transcribed into cDNA using M-MLV Reverse Transcriptase (TaKaRa, Japan). Real-time RT-PCR was performed using FastStart Universal SYBR Green Master (Roche, Germany). ST cells or IPEC-J2 cells were infected with TGEV or transfected with a plasmid encoding Nsp2. The NF-κB-specific inhibitor Bay 11–7082 (a pharmacological inhibitor of the IKK that phosphorylates IκBα [40]) or QNZ (a high-affinity antagonist of NF-κB pathway activation acting by inhibiting store-operated calcium (Ca^2+^) entry [41]) (Sigma, USA), was added to the media 1 h post-infection/post-transfection at a final concentration of 5 μM or 10 μM, respectively. Cells treated with DMSO served as a negative control. Expression of target genes mRNAs were measured 24 h post NF-κB-specific inhibitor treatment by real-time RT-PCR. The abundance of the individual mRNA transcripts in each sample was assayed three times and normalized to that of the expression of the housekeeping genes, GAPDH and β-actin, respectively. The primers used to detect Arginase and eNOS were from Reiner [42] and Zhu et al [43]. (shown in Table 2).

**Table 2.** Primers used for RT-PCR.

Gene name	Forward primer sequence (5ʹ-3ʹ)	Reverse primer sequence (5ʹ-3ʹ)
GAPDH	AAGGTCGGAGTGAACGGATTTG	GCCTTGACTGTGCCGTGGAAC
β-actin	GGTGGGTATGGGTCAGAAAG	TCCATGTCGTCCCAGTTGGT
IL-1β	AACGTGCAGTCTATGGAGT	GAACACCACTTCTCTCTTCA
IL-6	CTGCTTCTGGTGATGGCTACTG	GGCATCACCTTTGGCATCTT
IL-8	AGTTTTCCTGCTTTCTGCAGCT	TGGCATCGAAGTTCTGCACT
TNF-α	AACCTCAGATAAGCCCGTCG	ACCACCAGCTGGTTGTCTTT
Arginase	TTGCGAGACGTAGACCCTGG	CAAAGCTCAGGTGAATCGGC
eNOS	GCCTGAACAGCACAGGAGTT	ACGAGCAAAGGCACAGAAGT

### Virus growth curve

ST cells were infected with TGEV at an MOI of 0.1. After 1 h, NF-κB-specific inhibitor (Bay 11–7082 or QNZ) was added to the media at a final concentration of 5 μM or 10 μM. Cells treated with DMSO served as a negative control. Culture media were separately harvested at indicated time points from 12 h to 72 h. The virus titers of samples were determined using end-point dilution assays.

### Statistical analysis

All experiments were repeated a minimum of 3 times. The experimental data were statistically analyzed by two-way repeated measure ANOVA (RM-ANOVA) using Graphpad Prism software (version 5.0). P-values less than 0.05 were considered statistically significant, and those less than 0.01 were considered highly significant.
