# Supplementary figures and images for: Porcine transmissible gastroenteritis virus nonstructural protein 2 contributes to inflammation via NF-κB activation
Source: Virulence. 2018 Oct 29;9(1):1685–98. doi: 10.1080/21505594.2018.1536632 (PMC7000202; doi:10.1080/21505594.2018.1536632)

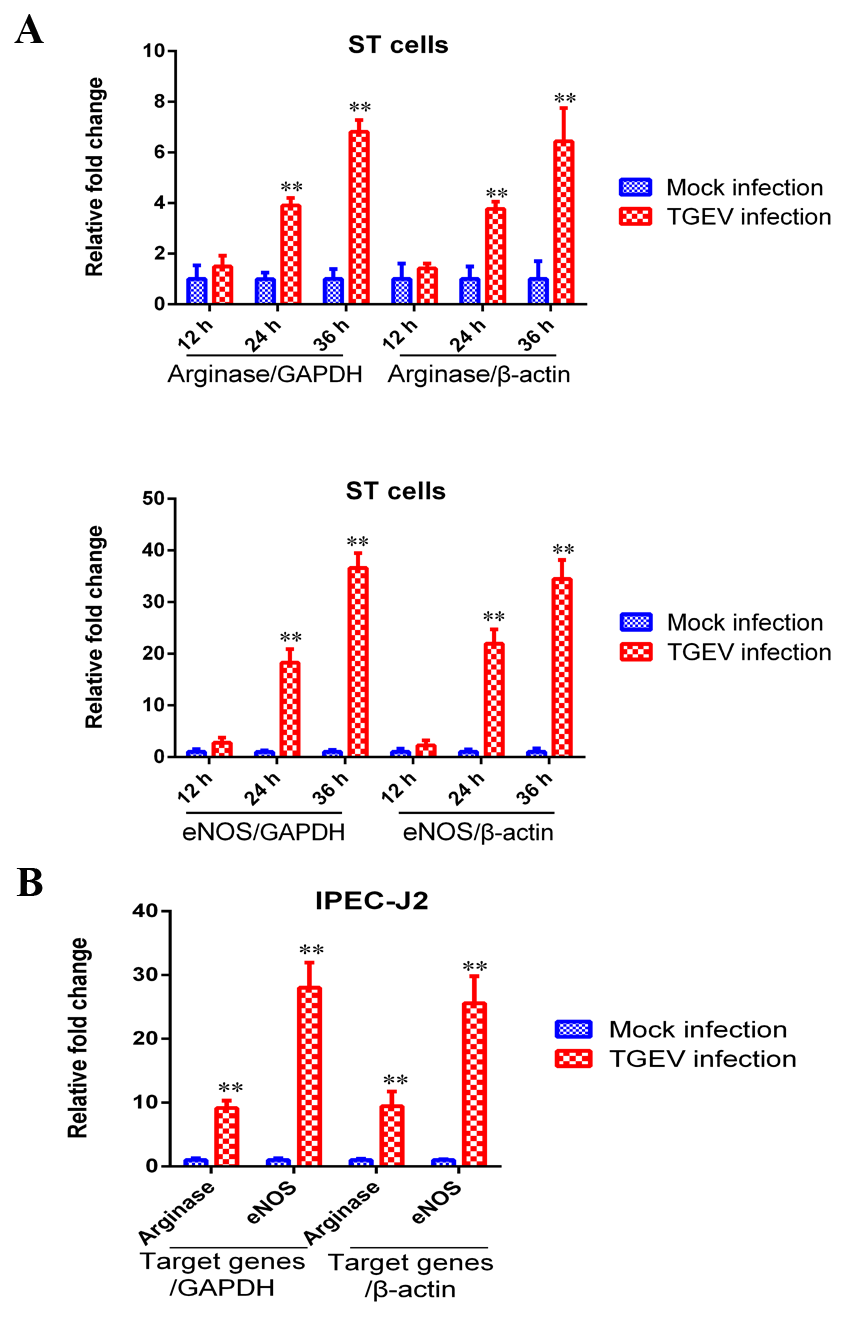

Supplement: Supplemental Material [file kvir-09-01-1536632-g000.zip › S1.tif]

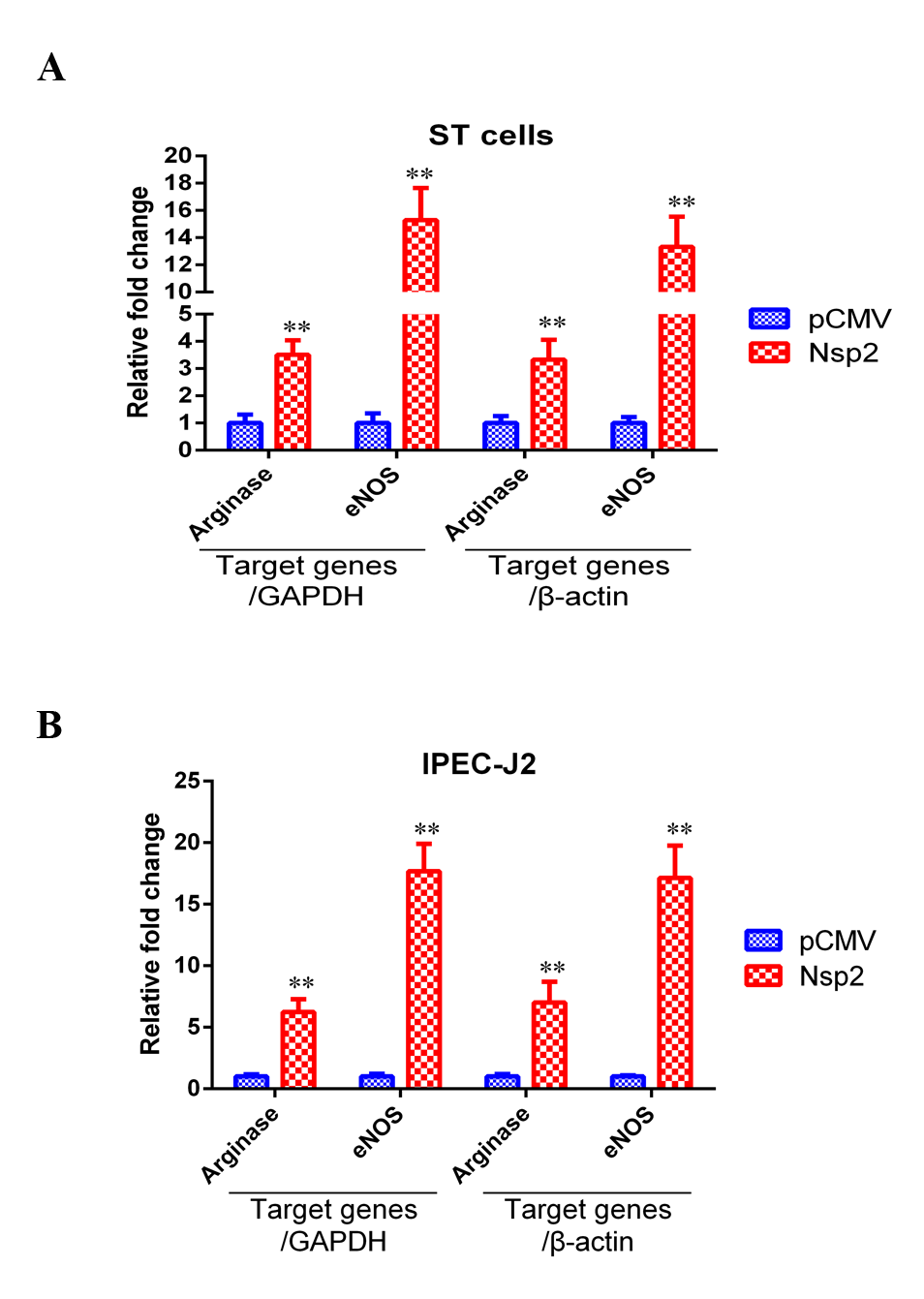

Supplement: Supplemental Material [file kvir-09-01-1536632-g000.zip › S2.tif]
